# Mixtures of Lipophilic Phycotoxins: Exposure Data and Toxicological Assessment

**DOI:** 10.3390/md16020046

**Published:** 2018-01-31

**Authors:** Jimmy Alarcan, Ronel Biré, Ludovic Le Hégarat, Valérie Fessard

**Affiliations:** 1 Toxicology of Contaminants Unit, French Agency for Food, Environmental and Occupational Health and Safety, ANSES, 35300 Fougères, France; jimmy.alarcan@anses.fr (J.A.); ludovic.lehegarat@anses.fr (L.L.H.); 2 Marine Biotoxins Unit, French Agency for Food, Environmental and Occupational Health and Safety, ANSES, 94706 Maisons-Alfort, France; ronel.bire@anses.fr

**Keywords:** phycotoxins, mixtures, exposure, toxicological assessment

## Abstract

Lipophilic phycotoxins are secondary metabolites produced by phytoplanktonic species. They accumulate in filter-feeding shellfish and can cause human intoxication. Regulatory limits have been set for individual toxins, and the toxicological features are well characterized for some of them. However, phycotoxin contamination is often a co-exposure phenomenon, and toxicological data regarding mixtures effects are very scarce. Moreover, the type and occurrence of phycotoxins can greatly vary from one region to another. This review aims at summarizing the knowledge on (i) multi-toxin occurrence by a comprehensive literature review and (ii) the toxicological assessment of mixture effects. A total of 79 publications was selected for co-exposure evaluation, and 44 of them were suitable for toxin ratio calculations. The main toxin mixtures featured okadaic acid in combination with pectenotoxin-2 or yessotoxin. Only a few toxicity studies dealing with co-exposure were published. In vivo studies did not report particular mixture effects, whereas in vitro studies showed synergistic or antagonistic effects. Based on the combinations that are the most reported, further investigations on mixture effects must be carried out.

## 1. Introduction

### 1.1. Problematic of Phycotoxins Contamination

Marine biotoxins are secondary metabolites produced by approximately 100 phytoplanktonic species [1]. From a chemical point of view, hydrophilic, lipophilic and amphiphilic toxins are distinguished. Among the group of lipophilic toxins, several main families have been depicted: okadaic acid (OA) and dinophysistoxins (DTXs), pectenotoxins (PTXs), yessotoxins (YTXs), azaspiracids (AZAs) and, finally, cyclic imines (spirolides (SPXs), pinnatoxins (PnTXs), pteriatoxins and gymnodimines (GYMs)). To prevent human intoxications, the European Union (EU) has set regulatory limits in shellfish [2] (Table 1).

However, several gaps exist in the current management of phycotoxins risk. For instance, no regulatory limits have been set up for cyclic imines, though these toxins are frequently detected and found to be very potent in vivo [3]. Regarding mixtures, the European Food Safety Authority (EFSA) opinion was only stated in the case of analogues based on toxicological equivalent factors (TEF) established from acute toxicity in rodents [2]. Although some publications reported the combined effects of a few binary mixtures of phycotoxins, a proper setting of regulation limits that would take into account risk when toxins co-occur is missing. Besides, it is noteworthy to investigate to which mixtures of phycotoxins the consumers can be exposed and to which respective levels. It is well known that some species can produce different analogues belonging to the same family, but also toxins of different families (Table 2). Moreover, as the conditions favoring the proliferation of deleterious phytoplankton, such as harmful algal bloom (HAB), can be similar for one species to another, several toxins are likely to co-occur.

### 1.2. Methodology for Mixture Hazard Assessment

**Figure 1:**
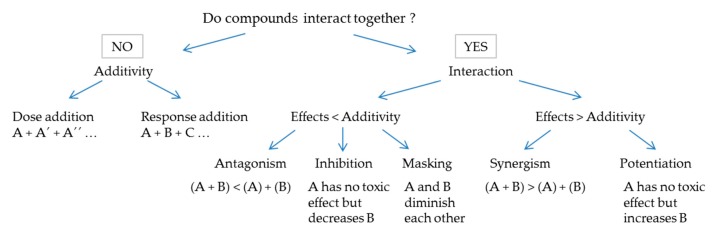
Methodology tree for mixture effect classification established according to the EFSA report [15].

Investigation of mixture effects is certainly one of the greatest challenges for hazard characterization nowadays. Hazard evaluation based on a single compound has restricted application since chemical contamination is often multiple and the interaction of compounds could result in a non-additive toxicity (whether higher or lower than expected). The combined effects of mixtures have been well established and classified [15]. This categorization relies on compounds sharing or not the same mode of action (MOA). Three different scenarios have been thus defined: (i) when compounds share the same MOA (analogues), the “dose addition” approach is employed: it considers that all these compounds behave as if they were a simple dilution of each other and the concentrations of each analogue are pondered using TEFs when available; (ii) when compounds have different MOAs, but no interaction is observed, the “response addition” approach is employed, and the global toxicity is calculated as the sum of each individual toxicity; (iii) when compounds interact, neither dose addition nor response addition are suitable approaches. Interaction is considered when the effect of a mixture differs from additivity based on the dose-response relationships of each individual compound. Then, effects are classified as lower (antagonism, inhibition, masking) or greater (synergism, potentiation) than additive. Figure 1 summarizes the different cases.

Such strategies have been successfully employed to characterize the mixture effects of pesticides, dioxins or heavy metals [16,17,18].

### 1.3. Toxicological Features of Phycotoxins

Okadaic acid and dinophysistoxins were first reported as responsible for diarrhetic shellfish poisoning (DSP), causing various symptoms in humans, such as diarrhea, nausea, abdominal pain or vomiting [19]. OA is a potent inhibitor of protein phosphatase 2A (PP2A) and to a lesser extent of PP1 [20]. The group of pectenotoxins, especially PTX-2, its main representative, used to be associated with DSP, but they were further removed from the diarrhetic toxins due to the lack of evidence for their implication in gastro-intestinal symptoms [21]. Nevertheless, according to the regulation, OA, DTXs and PTX-2 are summed together for the established limit of 160 µg of OA equivalent per kg of shellfish. The major deleterious effect of PTX-2 involves actin depolarization leading to cytoskeleton disruption [22]. The group of yessotoxins has not been reported to affect humans, but in vivo studies showed potent toxicity in rodents with intra-peritoneal administration and specific cardiotoxic effects with oral administration [23,24]. Many studies also claimed in vitro toxicity [25,26]. The mechanism of action is unknown, but YTX has been shown to interfere with the autophagy pathway [27]. Although the group of azaspiracids displays symptoms similar to DSP [28], in vivo studies in mice showed more severe effects than OA toxins [29]. AZAs were found to act as potassium channel blockers [30]. No food intoxication related to the group of cyclic imines has been reported so far. Still, cyclic imines have been shown to exert neurological effects in mice [31], and most spirolides including SPX-1 were shown to selectively inhibit nicotinic acetylcholine receptors [32].

## 2. Exposure Data

### 2.1. Case Study of Multi-Phycotoxins Contamination in Shellfish

For this review, we analyzed the literature dealing with multi-phycotoxins contamination using the Scopus and PubMed databases. One thousand one hundred seventy one references were retrieved from the Scopus database using the keywords dinophysistoxin, pectenotoxin, spirolide and yessotoxin. In PubMed, a total of 686 references was retrieved using the same keywords. Only studies including shellfish contamination with the different toxin-groups were considered for analysis excluding contamination data with different analogues of the same group (Table 3). Among these papers, only some were suitable for a case study analysis so as to estimate toxin ratios when co-exposure occurred. The papers for which toxin ratios were not reported or could not be determined were considered as unsuitable. The grey literature was not included in the search strategy, neither were the data collected from the national monitoring programs.

**Figure 2:**
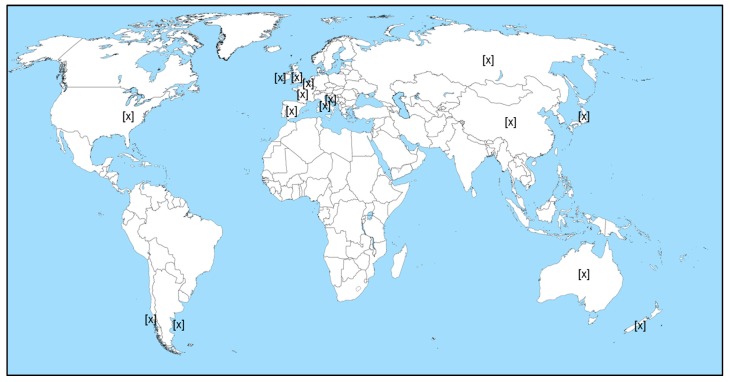
Case study of toxin-mixture contaminations. Countries where contaminations were reported are shown as [x]. A total of 44 publications considered as suitable was analyzed.

A total of 79 publications dealing with the co-occurrence of toxins in shellfish was retrieved. The mixtures reported depend on the toxins that were investigated. Table 3 sums the information on the toxin mixtures that were investigated in these studies. Among these 79 publications, only 44 were suitable for analysis. Geographical repartition is depicted in Figure 2.

According to Table 3, many studies did not investigate the presence of spirolides in shellfish. For instance, no data from the U.S., Japan or Korea were available. In Europe also, among the 36 references, 23 did not investigate the presence of spirolides. Similarly, the presence of azaspiracids or yessotoxins was not investigated in any of the studies.

When establishing a toxin ratio A/B, A always corresponds to the toxin found in the highest concentration. For instance, in their paper, Pavela-Vrancic et al., 2002 [65], reported 0.133 and 0.090 µg/g hepato-pancreas (HP) of OA and 7-epi-PTX-2SA, respectively. Therefore, the ratio OA/7-epi-PTX-2SA equals 1.5 (= 0.133/0.090). When multiple analogues of the same toxin-group were reported, they were arithmetically summed without taking into account TEF values when available and named as equivalent to the corresponding toxin leader (OA, PTX-2, AZA-1, YTX and SPX-1). This choice was made to circumvent the fact that TEFs are not available for all of the toxins. Furthermore, one cannot be sure that the TEFs would still be valid for mixtures of toxins belonging to different groups. For instance, data for OA, DTX-1 and DTX-2 were summed and called OA equivalent (OA eq.). The complete and detailed analysis of each publication is supplied in the Supplementary Data Table S1.

**Figure 3:**
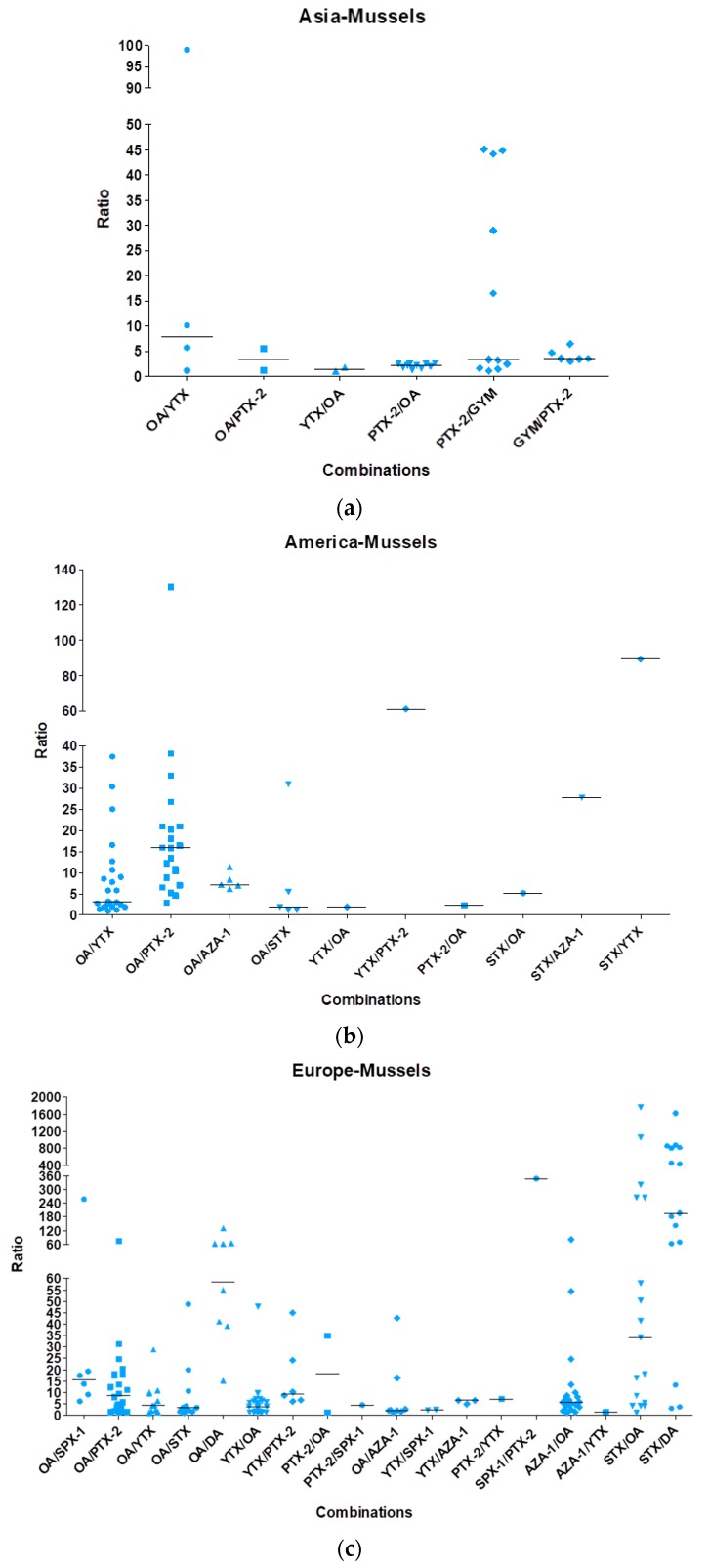

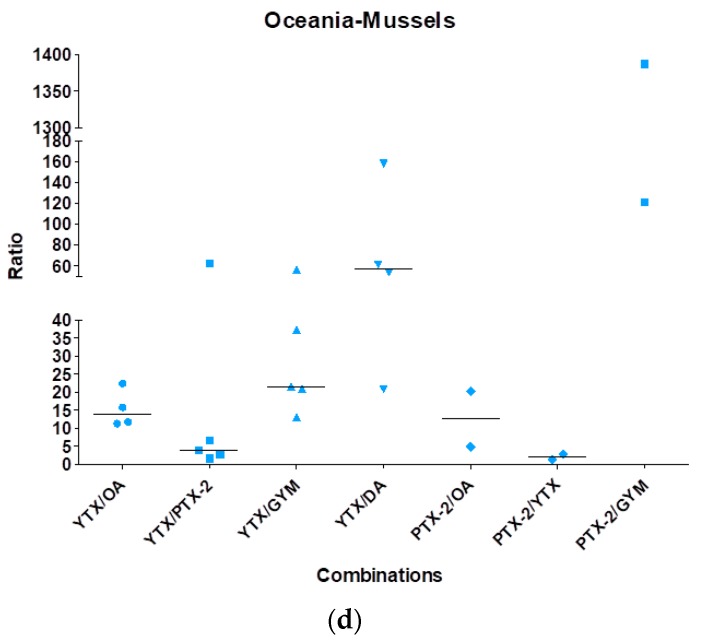
Mixture ratios found in mussels based on the analysis of 44 publications. (**a**) Data for Asia, (**b**) for America, (**c**) for Europe and (**d**) for Oceania.

**Figure 4:**
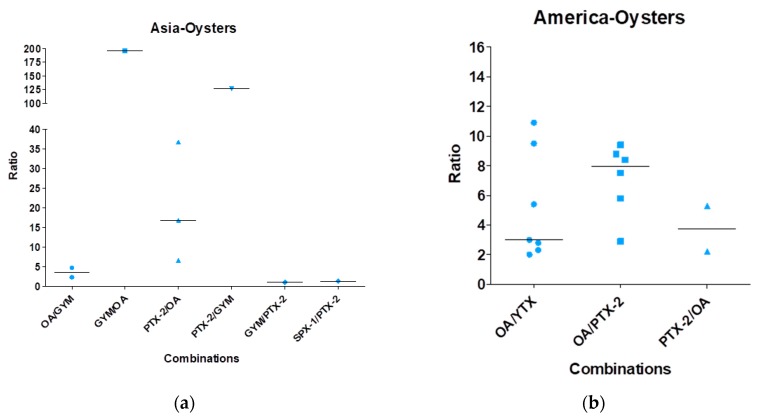

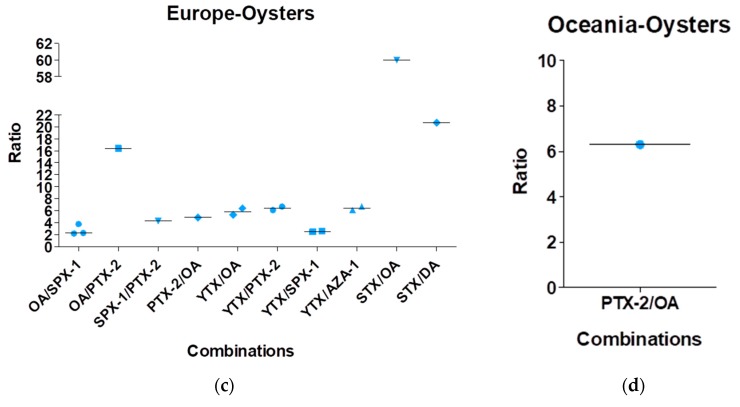
Mixture ratios found in oysters based on the analysis of the 44 publications. (**a**) Data for Asia, (**b**) for America, (**c**) for Europe and (**d**) for Oceania.

**Figure 5:**
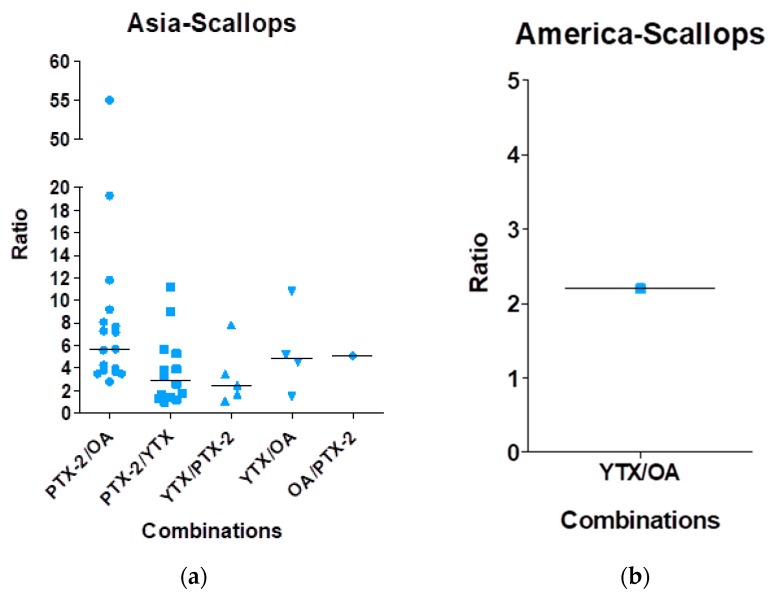

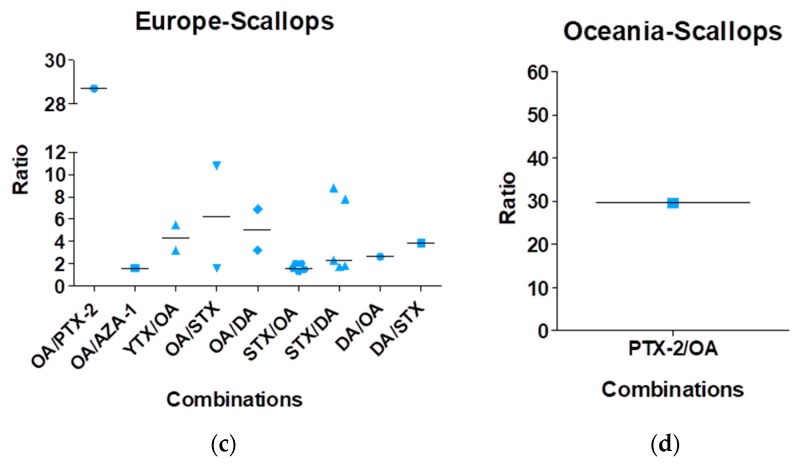
Mixture ratios found in scallops based on the analysis of the 44 publications. (**a**) Data for Asia, (**b**) for America, (**c**) for Europe and (**d**) for Oceania.

**Figure 6:**
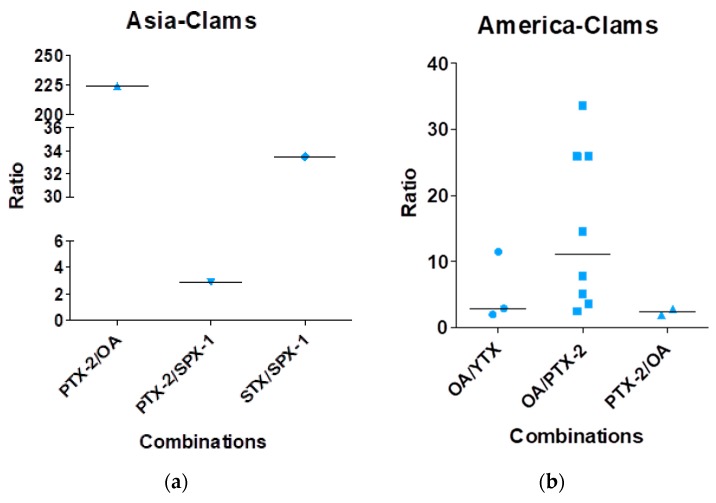

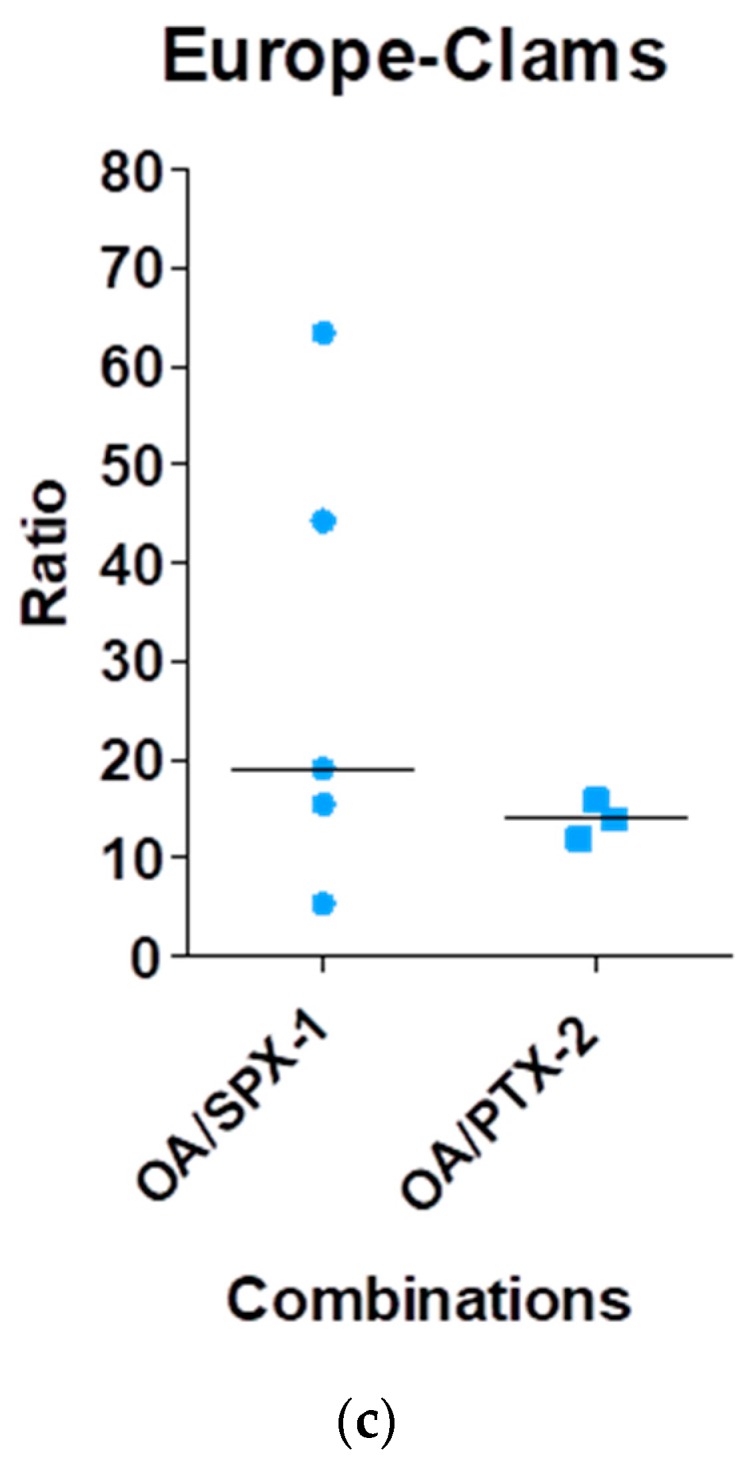
Mixture ratios found in clams based on the analysis of the 44 publications. (**a**) Data for Asia, (**b**) for America and (**c**) for Europe.

From these analyses, it appears that OA was the most often recorded lipophilic toxin in mixtures, as well as the predominant toxin (amount) whatever the mixture. Binary and trinary mixtures were also reported and sometimes even more complex cocktails (up to five toxins). In order to give a global view of mixtures, data from all publications were compiled and gathered according to shellfish species and geographic localization (Figure 3, Figure 4, Figure 5 and Figure 6). Data in Figure 3, Figure 4, Figure 5 and Figure 6 depict only the ratios for binary combinations. For instance, a trinary mixture OA/YTX/SPX-1 (OA being the predominant toxin) is represented by two dots considering the predominant toxin: one dot for OA/YTX and the other for OA/SPX-1. For each binary combination, the toxin ratios and their median values were calculated and presented by dots and horizontal lines, respectively. Different patterns were used to depict data and are solely meant to ease the reading of the figures, without specific correspondences.

Figure 3 shows the data regarding the contamination of mussels. In Asia, six combinations were reported: OA/YTX with a median ratio of eight and all the other combinations (OA/PTX-2, YTX/OA, PTX-2/OA, PTX-2/GYM and GYM/PTX-2) with a median ratio between one and five. In America, ten combinations were reported: OA/YTX, OA/STX, YTX/OA, PTX-2/OA with similar median ratios of 3–4, OA/PTX-2 with a median ratio of 15, OA/AZA-1 and STX/OA with median ratios between 5 and 8, STX/AZA-1 with a ratio around 28, YTX/PTX-2 with a ratio of 60, and STX/YTX with a ratio around 90. In Europe, 18 combinations were reported: OA/SPX-1 and PTX-2/OA with similar median ratios of 16, OA/PTX-2 and YTX/PTX-2 with similar median ratios of 8–9, STX/OA with a median ratio of 33, OA/DA with a median ratio of 58, STX/DA with a median ratio of 200, SPX-1/PTX-2 with a ratio of 350 and all other combinations (OA/YTX, OA/STX, YTX/OA, PTX-2/SPX-1, OA/AZA-1, YTX/SPX-1, YTX/AZA-1, PTX-2/YTX, AZA-1/OA and AZA-1/YTX) with median ratios between 1 and 7. In Oceania, seven combinations were reported: YTX/PTX-2 and PTX-2/YTX with median ratios between 1 and 4, YTX/OA and PTX-2/OA with similar median ratios of 13–14, YTX/GYM with a median ratio of 21, YTX/DA with a median ratio of 60 and PTX-2/GYM with a median ratio around 750.

Figure 4 shows the data regarding the contamination of oysters. In Asia, six combinations were reported: OA/GYM, GYM/PTX-2 and SPX-1/PTX-2 with ratios between 1 and 4, PTX-2/OA with a median ratio of 16, PTX-2/GYM with a ratio of 125 and GYM/OA with a ratio around 200. In America, three combinations were reported: OA/YTX and PTX-2/OA with similar median ratios of 3–4 and OA/PTX-2 with a median ratio of eight. In Europe, 10 combinations were reported: OA/PTX-2 with a ratio around 16, STX/DA with a ratio of 21, STX/OA with a ratio of 60 and all the other combinations (OA/SPX-1, SPX-1/PTX-2, PTX-2/OA, YTX/OA, YTX/PTX-2, YTX/SPX-1 and YTX/AZA-1) with a median ratio between 2 and 6. In Oceania, only the mixture PTX-2/OA with a ratio of six was reported.

Figure 5 shows the data regarding the contamination of scallops. In Asia, five combinations were reported: PTX-2/YTX and YTX/PTX-2 with similar median ratios of 3, YTX/OA and OA/PTX-2 with similar median ratios of 5 and PTX-2/OA with a median ratio of 6. In America, only the mixture YTX/OA with a ratio around two was reported. In Europe, nine combinations were reported: OA/PTX-2 with a ratio of 29 and all the other combinations (OA/AZA-1, YTX/OA, OA/STX, OA/DA, STX/OA, STX/DA, DA/OA and DA/STX) with a median ratio between 2 and 6. In Oceania, only the mixture PTX-2/OA with a ratio around 30 was reported.

Figure 6 shows the data regarding the contamination of clams. In Asia, three combinations were reported: PTX-2/SPX-1 with a ratio of 3, STX/SPX-1 with a ratio of 34 and PTX-2/OA with a ratio of 225. In America, three combinations were reported: OA/YTX and PTX-2/OA with similar median ratios of three and OA/PTX-2 with a median ratio of 11. In Europe, two combinations were reported: OA/PTX-2 with a median ratio of 13 and OA/SPX-1 with a median ratio of 20. No mixtures were reported in Oceania in this particular matrix.

**Figure 7:**
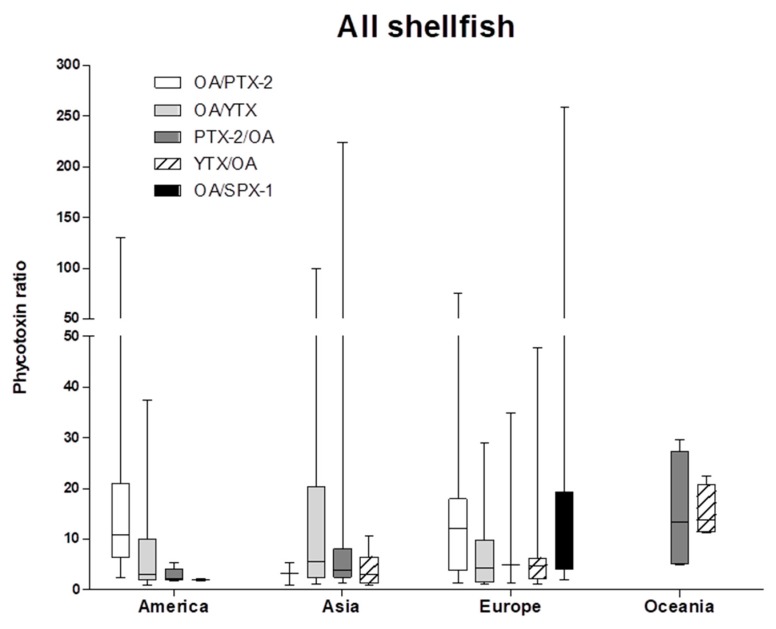
Box and whisker plots of phycotoxins ratios calculated for the main reported mixtures according to the location. The minimum, the lower quartile, the median, the upper quartile and the maximum are shown in the box and whisker plots.

From our cases study, it appears that shellfish contamination by mixtures depends on the location. For instance, mixtures involving SPX-1 were often reported in Europe and in several shellfish types (mussel, oyster, clam, scallop and cockle), whereas it was scarcely described in Asia. In fact, in Japan and Korea, neither SPXs, nor AZAs were investigated. In Oceania, OA was found to be minor in mixtures, whereas it was predominant in mixtures reported in Europe and America. As for the ratios, Figure 7 shows box plots for the main reported combinations. Except in Asia, the median value ratio for the combination OA/PTX-2 is superior to 10 and higher in Europe compared to America. The median value ratio for the combination OA/YTX is around 3.5, except in Asia, where it yields six. For the combination OA/SPX-1, it reaches 11.5, but this combination is only reported in Europe. The combinations PTX-2/OA and YTX/OA share a similar value of the median ratios for a defined zone, but these ratios are continent-dependent (around 2 for America, 4–5 in Europe and 14 in Oceania). In Asia, median values ratios for PTX-2/OA and YTX/OA combinations are around 3–4. Besides, data also show that the distribution of the ratio values can be very wide for some combinations, with an upper extreme value more than 10-times higher than the median value for other combinations.

Regarding the other publications that describe multi-toxins contamination, but which were not selected for the case study, the information is reported in Table 3. In Africa, most of the data concern Morocco. The main mixtures featured OA, DTXs and AZAs. In America, the mixtures featured often OA or DTX-1 with PTX-2, YTX and traces of spirolides and AZAs. In Asia, OA was found predominantly in association with PTX-2. In Europe, the main mixtures featured OA, DTXs and PTX-2 or YTX.

### 2.2. Multi-Phycotoxins Contamination in Other Matrices

Throughout our literature analysis, we found some papers describing multi-phycotoxin contamination in matrices other than shellfish (Table 4). Most of the time, the matrix was gastropods. Compared to shellfish, new combinations were described such as OA/PnTXs, OA/ciguatoxin (CTX) or OA/DA/Brevetoxin 3 (PbTx-3).

### 2.3. Conclusions and Perspectives Regarding Multi-Phycotoxins Contamination in Shellfish

Multi-phycotoxins contamination of seafood has been detected worldwide. The variability of analogues and bivalve filtering species, as well as discrepancies between geographical areas make it very challenging to establish a proper picture of multi-toxin contamination. From our literature analysis, it appears that the most frequent mixtures imply OA in combination with PTX-2 or YTX. If OA/PTX-2 mixtures depicted a median value ratio superior to 10 in America and Europe, a lower median ratio (inferior to five) was observed for PTX-2/OA mixtures. On the contrary, OA/YTX and YTX/OA mixtures share a similar ratio-value (around 3–4). Finally, even if OA/SPX-1 was only reported in Europe with a median value ratio of 11.5, the occurrence of this mixture could be underestimated since SPX-1 was not often included in the monitoring of non-European countries. In our review, the focus was on lipophilic toxins, but mixtures of both lipophilic and hydrophilic toxins have been also observed in a few cases. As depicted in Table 3, many studies did not investigate the presence of toxins such as spirolides, azaspiracids and even sometimes yessotoxins. Consequently, some of the mixtures that were described may not be fully accurate. For the purposes of this work, the toxins belonging to the same group were expressed as the equivalent of the main analogue. Besides, all the mixtures featuring more than two compounds were converted into binary mixtures. Most of the data were obtained from shellfish sampling in a short period that does not reflect any seasonal variability. In order to improve toxin mixtures’ identification, it could be worth creating a network to analyze phycotoxin contamination with a shared database between institutes in charge of toxin monitoring. The better our knowledge on data exposure, the better we will be able to assess mixture effects. Indeed providing sufficient exposure data will enable selecting the most relevant mixtures (concentrations and ratios) before performing in vitro and in vivo assays, especially as in vivo investigations are toxin and money-consuming.

## 3. Toxicological Assessment

### 3.1. In Vivo Studies

So far, only a few studies have been conducted regarding possible mixture effects. Two of them consisted of one single dose treatment, whereas a third one mimicked a short-term repeated exposure. For all studies, the oral route was the way of administration. Table 5 summarizes the experimental conditions and the results.

In the study of Aasen et al. [118], female NMRI mice were given by gavage 1 or 5 mg/kg YTX, either alone or together with 200 mg/kg AZA-1. The results indicated no particular mixture effects in regards to clinical effects and pathological changes of internal organs. However, an increase in YTX levels was observed in stomach tissue suggesting higher YTX absorption in stomach when YTX was combined with AZA-1. After determination of the lethal doses of OA or AZA-1 by gavage to female NMRI mice, Aune et al. [119] examined the combined toxicity of OA and AZA-1 when given at both LD_10_ and LD_50_/LD_10_ doses. No combined effects on lethality when AZA-1 and OA were given together were reported. Similarly, the pathological effects along the gastro-intestinal tract were not increased. The absorption of OA and AZA-1 from the GI tract was very low for each toxin separately, and it was reduced when toxins were given together. The in vivo toxicity by repeated oral exposure to a combination of YTX and OA (1 mg YTX/kg and 0.185 mg OA/kg, daily for seven days) was investigated in female CD-1 mice [120]. The results indicated no mortality, signs of toxicity, diarrhea and hematological changes, neither with the toxins alone, nor when co-administration. Thus, the co-exposure of YTX and OA did not show any combined toxic effects in mice. Franchini et al., 2005 [121], also featured mixtures of toxins (OA/YTXs), but since the effects of YTXs alone were not investigated, it is not possible to conclude about any mixture effect.

### 3.2. In Vitro Studies

Data concerning in vitro effects of toxins mixtures are scarce. Nevertheless, it has been pinpointed that a combination of toxins can result in greater or lower toxicity compared to toxins alone. For example, Sala et al., 2009 [122], showed a synergistic effect on the protein expression of heat shock protein β-1 isoforms and superoxide dismutase in human breast adenocarcinoma cells after 24 h of co-treatment with OA and gambierol (50/50 nM). Nonetheless, the characterization of those interactions using a mathematical model is missing in order to fully conclude about a mixture effect.

Ferron et al., 2016 [123], used the combination index-isobologram equation developed by Chou and Talalay [124] in order to deeply characterize the interactions between binary mixtures of phycotoxins incubated with human intestinal cells (Table 6).

All kinds of mixture effects, i.e., synergism, additivity and antagonism, were depicted in this study. Although Rodriguez et al. [103] showed a greater toxicity in human neuroblastoma cells when OA was co-incubated with YTX or DTX-2, only an additive effect could be concluded from their results, as they did not take into account the additivity of the effects.

### 3.3. Conclusions and Perspectives Regarding Multi-Phycotoxins’ Toxicological Assessment

Except some modification in the absorption of toxins, no particular in vivo combined effects have been depicted so far. On the contrary, in vitro studies reported synergism, antagonism and additivity. Interestingly, the mixtures that failed to induce any in vivo combined effects were potent on cell lines. At least one of the most common mixtures OA/YTX showed a panel of responses from antagonism to synergism depending on the molar ratios. In vitro models are certainly the most suitable tools for screening combined effects as a large range of toxins concentrations and ratios can be investigated. Surprisingly, no in vivo studies featuring mixtures of OA/SPX-1 and OA/PTX-2 were conducted, although these two combinations were commonly found in contaminated seafood.

## 4. Conclusions

The purpose of this review was to summarize the knowledge about published data dealing with seafood contamination by mixtures of lipophilic phycotoxins. Since mixtures can modulate the toxicity, the combined effects are worth investigating to identify the mixtures with higher potencies that may affect human health. For this purpose, relevant combinations (toxin composition and ratios between the toxins) must be established before performing toxicological surveys. As stated before, giving a complete overview of the occurrence of phycotoxins mixtures is challenging. Nevertheless, this review points out which combinations were most reported in the literature and which ratios were displayed. Additional data on mixtures of lipophilic phycotoxins, both on exposure and on toxicity, are required to state if the current regulations are sufficient and relevant to protect consumers’ health. 

## Figures and Tables

**Table 1:** Current EU limits, exposure levels resulting from consumption of shellfish on the EU market and acute reference doses (ARfDs) set by the European Food Safety Authority (EFSA) (taken from EFSA Report #1306, [2]).

Toxin Group	Current EU Limits in Shellfish Meat	Exposure by Eating a 400-g Portion at the EU Limit	ARfD
OA and analogues	160 µg OA eq./kg SM	64 µg OA eq./person	0.3 µg OA eq./kg b.w.
AZA	160 µg AZA eq./kg SM	64 µg AZA1 eq./person	0.2 µg AZA1 eq./kg b.w.
PTX	160 µg OA eq./kg SM	64 µg PTX2 eq./person	0.8 µg PTX2 eq./kg b.w.
YTX	1 mg YTX eq./kg SM	400 µg YTX eq./person	25 µg YTX eq./kg b.w.
STX	800 µg PSP/kg SM	320 µg STX eq./person	0.5 µg STX eq./kg b.w.
DA	20 mg DA/kg SM	8 mg DA/person	30 µg DA/kg b.w.

SM: shellfish meat; eq.: equivalents; b.w.: body weight; ARfD: acute reference dose; PSP: paralytic shellfish poison; EU: European Union; OA: okadaic acid; PTX: pectenotoxin; YTX: yessotoxin; STX: saxitoxin; DA: domoic acid; AZA: azaspiracid.

**Table 2:** Global overview of the key phytoplanktonic species producing the main lipophilic phycotoxins. SPX, spirolide.

Phycotoxins	Species	Ref.
OA/DTXs	*Dinophysis mitra*, *Dinophysis tripos*, *Prorocentrum lima*, *Prorocentrum concavum*	[4,5]
OA/DTXs and PTXs	*Dinophysis fortii*, *Dinophysis acuta*, *Dinophysis acuminata*, *Dinophysis norvegica*, *Dinophysis rotundata*	[4,6,7,8,9]
YTXs	*Protoceratium reticulatum*, *Lingulodinium polyedrum*, *Gonyaulax spinifera*	[10,11]
AZAs	*Azadinium spinosum*	[12]
SPXs	*Alexandrium ostenfeldii*, *Alexandrium peruvianum*	[13,14]

**Table 3:** List of publications where multi-phycotoxins contamination in shellfish were reported. Red color indicates that the data were unsuitable for analysis.

Authors	Ref.	Area	Toxins Investigated	Toxins Mixtures Reported
Taleb et al., 2006	[33]	Morocco	OA, DTX-1, DTX-2, AZA-1, AZA-2, AZA-3	mixtures of OA, DTX-2, AZA-2 and AZA-1
Elgarch et al., 2008	[34]	Morocco	OA, DTX-1, DTX-2, AZAs	mixtures of OA, DTX-2 and traces of AZA-2. OA found in highest concentrations
Ben Haddouch et al., 2015	[35]	Morocco	OA, DTXs, PTXs, AZAs, GYMs, SPXs, YTXs	mixtures of OA, DTXs, YTX, PTXs, AZA-2 and sometimes GYM
Pitcher et al., 2011	[36]	South Africa	OA, DTX-1, DTX-2, PTXs, AZA-1, GYM, SPXs, YTX, DA	mixtures of OA, DTX-1 and traces of PTXs
Turner et al., 2015	[37]	Argentina	OA, DTX-1, DTX-2, PTX-1, PTX-2, PTX-11, AZA-1, AZA-2, AZA-3, GYM, SPX-1, 20 Me SPX-G, YTX, 45-OH-YTX, homoYTX, 45-OH-homoYTX	YTX/OAs
McCarron et al., 2014	[38]	Canada	DA, OA, DTXs, AZAs, PTXs, YTXs, GYMs, SPXs, PnTXs.	mixtures of high levels of DTX-1, PTXs, YTXs and trace levels of cyclic imines
Alvarez et al., 2010	[39]	Chile	OA, DTX-1, PTX-1, PTX-2, PTX-2 sa, AZA-1, SPX-1, YTX	mixtures of AZA-1 and SPX-1; levels were below LOQ
Garcia et al., 2012	[40]	Chile	OA, DTX-1, DTX-2, PTX-2, YTX, AZA-1	DTX-1/PTX-2/YTX
Zamorano et al., 2013	[41]	Chile	OA, DTX-1, DTX-2, PTX-2, AZA-1, AZA-2, AZA-3, YTX, STX, neo-STX, GTXs	OAs/PTX-2/AZA-1/YTX/STXs
Alves de Souza et al., 2014	[42]	Chile	OA, DTX-1, DTX-2, DTX-3, PTX-2, YTX, 45-OH-YTX	mixture of 45-OH-YTX and traces of PTX-2
García et al., 2015	[43]	Chile	OA, DTX-1, DTX-2, PTX-2, AZA-1, AZA-2, AZA-3, YTX, STX, neo-STX, GTXs	mixtures of STXs and OA/DTX-1; hydrophilic toxins were subjected to shellfish metabolism
Garcia et al., 2016	[44]	Chile	OA, DTX-1, DTX-2, DTX-3, PTX-2, PTX-2 sa, AZA-1, AZA-2, AZA-3, YTX, homoYTX, COOH-YTX	OAs/PTX-2/YTX and OAs/YTX
García-Mendoza et al., 2014	[45]	Mexico	OA, DTX-1, DTX-2, PTX-1, PTX-2, PTX-11, AZA-1, AZA-2, AZA-3, GYM, SPX-1, YTX, 45-OH-YTX, homoYTX, 45-OH-homoYTX	mixtures mainly of OA, PTX-2, YTX and low levels of SPX-1 and AZA-1
Trainer et al., 2013	[46]	U.S.	OA, DTX-1, DTX-2, PTX-2, AZA-1, AZA-2, AZA-3, YTX	OA/YTX/PTX-2 and OA/PTX-2 and OA/YTX and OA/PTX-2/AZA-2 and OA/YTX/PTX-2/AZA-2
Hattenrath-Lehmann et al., 2013	[47]	U.S.	OA, DTX-1, DTX-2, PTX-2, PTX-11	OAs/PTXs
Eberhart et al., 2013	[48]	U.S.	OA, DTX-1, DTX-2, YTX	mixtures of DTX-1 and YTX
Wu et al., 2005	[49]	China	OA, DTX-1, STX, neo-STX, GTXs	mixtures of OA and GTX-2/3
Liu et al., 2011	[50]	China	OA, DTX-1, DTX-2, PTX-1, PTX-2, AZA-1, AZA-2, AZA-3, GYM, SPX-1, SPX-A, YTX, 45-OH-YTX, homoYTX, 45-OH-homoYTX	GYM/OA and PTX-2s/OA
Li et al., 2012	[51]	China	OA, DTX-1, DTX-2, PTX-2, PTX-2 sa, AZA-1, AZA-2, AZA-3, GYM, SPX-1, YTX, 45-OH-YTX	OAs/PTX-2s
Guo et al., 2012	[52]	China	OA, DTX-1, PTX-2, YTX	OAs/PTX-2
Zhang et al., 2012	[53]	China	OA, DTX-1, PTXs	mixture of OA, DTX-1, 7-epi-PTX-2sa and PTX-2sa
Li et al., 2014	[54]	China	OA, DTX-1, DTX-3, PTXs, AZA-1, AZA-2, AZA-3, GYM, SPX-1, YTX	PTX-2s/GYM and PTX-2s/GYM/OAs and PTX-2s/OAs
Fang et al., 2014	[55]	China	PTX-2, AZA-2, GYM, SPX-1	SPX-1/PTX-2
Wu et al., 2014	[56]	China	OA, DTX-1, DTX-2, PTX-2, AZA-1, AZA-2, AZA-3, GYM, SPX-1, YTX, PbTXs	mixtures of OA, SPX-1, PTX-2, AZAs, PbTx-3 and traces of YTX
Wang et al., 2015	[57]	China	OA, DTX-1, DTX-2, PTX-2, AZA-1, AZA-2, AZA-3, GYM, SPX-1, YTX	mixtures of OA, DTX-1, PTX-2 and GYM
Wu et al., 2015	[58]	China	OA, PTX-2, AZA-1, GYM, SPX-1	OA/PTX-2/GYM/SPX-1 and OA/AZA-1/PTX-2/GYM/SPX-1 and OA/PTX-2/GYM
Li et al., 2016	[59]	China	OA, DTX-1, PTXs, AZA-1, AZA-2, AZA-3, GYM, SPX-1, YTXs	STXs/SPXs/YTXs and PTX-2/SPXs and STX/SPXs and OA/didesMe-SPX-C
Jiang et al., 2017	[60]	China	OA, DTX-1, DTX-2, PTX-1, PTX-2, PTX-2 sa, AZA-1, AZA-2, AZA-3, GYM, SPX-1, YTXs, DA	PTX-2s/OA/GYM and DTX-1/GYM
Suzuki et al., 2000	[61]	Japan	OA, DTX-1, PTX-6	PTX-6/OAs
Ito et al., 2001	[62]	Japan	OA, DTX-1, PTX-6, YTX	mixtures constituted of OA, DTX-1, YTX and PTX-6
Suzuki et al., 2005	[63]	Japan	OA, DTX-1, DTX-2, PTXs, YTXs	PTX-2s/OAs/YTXs and OAs/YTXs
Hashimoto et al., 2006	[64]	Japan	OA, DTX-1, DTX-3, PTX-1, PTX-2, PTX-6, YTX, 45-OH-YTX	PTX-2s/YTXs/OAs
Suzuki et al., 2011	[65]	Japan	OA, DTX-1, DTX-2, PTXs, YTXs	PTX-2s/OAs/YTXs and OAs/YTXs
Matsushima et al., 2015	[66]	Japan	OA, DTX-1, DTX-3, PTX-1, PTX-2, PTX-3, PTX-6	mixtures mainly of PTX-6 and DTX-3
Kim et al., 2010	[67]	Korea	OA, DTX-1, PTX-2, YTX	mixtures of OA, DTX-1 and traces of PTX-2, YTX
Lee et al., 2011	[68]	Korea	OA, DTX-1, PTX-2, YTX	mixtures mainly constituted of OA and DTX-1; DSP toxin content 10-times higher in mussels than in oysters
Vershinin et al., 2006	[69]	Russia	OA, DTX-1, PTXs, YTXs, AZAs, SPX-1	OAs/PTXs/YTXs
Morton et al., 2009	[70]	Russia	OA, DTX-1, PTXs	mixtures of OA, DTX-1, PTX-2 and PTX-2 sa
Orellana et al., 2017	[71]	Belgium	OA, DTX-1, DTX-2, PTX-2, AZA-1, AZA-2, AZA-3, SPX-1, YTX	mixtures of OA, DTX-2, SPXs and their ester metabolites
Pavela-Vrancic et al., 2001	[72]	Croatia	OA, DTX-2, PTX-2 sa, 7-epi-PTX-2 sa	mixtures of OA and 7-epi-PTX-2sa
Pavela-Vrancic et al., 2002	[73]	Croatia	OA, DTX-1, DTX-2, PTX-2, PTX-2 sa, 7-epi-PTX-2 sa	OA/7-epi-PTX-2SA
Pavela-Vrancic et al., 2006	[74]	Croatia	OA, DTX-1, DTX-2, PTX-2 sa, 7-epi-PTX-2 sa	OA/7-epi-PTX-2SA
Ninčević Gladan et al., 2008	[75]	Croatia	OA, DTX-1, DTX-2, PTX-2, PTX-2 sa, PTX-6, AZAs, GYM, SPX, YTX, COOHYTX, 45-OH-YTX, homoYTX, 45-OH-homoYTX	YTXs/OA and OA/YTXs
Ninčević Gladan et al., 2010	[76]	Croatia	OA, DTX-1, DTX-2, PTXs, YTXs, GYM, SPX-1	YTXs/OA and OA/YTXs/PTX-2s and OA/PTX-2s
Čustović et al., 2014	[77]	Croatia	OA, DXT-3, YTX, PSP	YTX/OAs
Amzil et al., 2007	[78]	France	OA, DTX-1, DTX-2, DTX-3, PTXs, AZAs, YTXs, SPXs, GYMs	OA/PTX-2/SPXs and OA/SPXs and PTX-2/OA
Amzil et al., 2008	[79]	France	OA, DTXs, PTXs, PTX-6, AZAs, GYMs, SPXs, YTXs	mixtures of OA, AZA-1 and AZA-2
Picot et al., 2012	[80]	France	OA, SPX-1	OA/SPX-1
Fernandez Puente et al., 2004	[81]	Ireland	OA, DTX-1, DTX-2, PTX-2, PTX-2 sa	OAs/PTX-2s
Fux et al., 2009	[82]	Ireland	OA, DTX-1, DTX-2, PTX-2, YTX, SPX, AZA-1, AZA-2, AZA-3	AZAs/OAs and OAs/AZAs and OAs/AZAs/YTX
Campbell et al., 2014	[83]	Ireland	OA, DTX-1, DTX-2, DA, STX, palytoxin	PSP/OAs/DA
Ciminiello et al., 1997	[84]	Italy	OA, YTX	YTX/OA
Draisci et al., 1999	[85]	Italy	OA, YTX, homoYTX	OA/YTX
Draisci et al., 1999	[86]	Italy	OA, DTX-1, DTX-2, PTXs, YTX	mixture of YTX, PTXs and OA
Ciminiello et al., 2010	[87]	Italy	OA, DTX-1, DTX-2, PTXs, AZA-1, AZA-2, AZA-3, YTXs, SPXs, DA	SPXs/PTX-2sa
Nincevic Gladan et al., 2011	[88]	Italy	OA, DTX-1, DTX-2, PTX-1, PTX-2, PTX-2 sa, 7-epi-PTX-2 sa, PTX-6, GYM, SPX-1, YTX, 45-OH-YTX, homoYTX, 45-OH-homoYTX	OA/homoYTX and OA/homoYTX/PTX-2sa and OA/PTX-2sa
Buratti et al., 2011	[89]	Italy	OA, YTX, 45-OH-YTX, homoYTX, COOH-YTX	mixtures mainly of YTX and homoYTX. HomoYTX found in highest concentrations
Bacchiocchi et al., 2015	[90]	Italy	OA, DTX-1, DTX-2, PTX-1, PTX-2, AZA-1, AZA-2, AZA-3, YTX, 45-OH-YTX, homoYTX, 45-OH-homoYTX	mixtures mainly of OA and YTX plus traces of AZA-2
Gerssen et al., 2010	[91]	The Netherlands	OA, PTX-2, AZA-1, YTX, SPX-1	YTX/OA/AZA-1/PTX-2/SPX-1
Van den Top et al., 2011	[92]	The Netherlands	OA, DTX-1, DTX-2, PTX-2, AZA-1, AZA-2, AZA-3, YTX, 45-OH-YTX	OAs/AZAs/YTXs/PTX-2 and YTXs/OAs and YTXs/OAs/AZAs
Gerssen et al., 2011	[93]	The Netherlands	OA, DTXs, PTXs, AZAs, YTXs	OAs/AZAs/PTX-2s and OAs/AZAs/YTXs/PTX-2s and PTX-2s/OAs/YTXs
Lee et al., 1988	[94]	Norway	OA, DTX-1, PTX-2, YTX	mixtures of DTX-1 and YTX
Ramstad et al., 2001	[95]	Norway	OA, DTX-1, YTX	mixtures constituted of OA/DTX-1 and YTX
Torgersen et al., 2008	[96]	Norway	OA, DTXs, PTXs	mixtures of PTXs, OA and DTXs
Vale et al., 2004	[97]	Portugal	OA, DTX-1, DTX-2, PTX-2, PTX-2 sa, 7-epi-PTX-2 sa	mixtures of OA/DTX-2 and PTX-2/PTX-2sa
Vale et al., 2006	[98]	Portugal	OA, DTX-1, DTX-2, PTX-2, PTX-2 sa, 7-epi-PTX-2 sa	mixtures of OA/DTX-2 and PTX-2/PTX-2sa
Gago-Martinez et al., 1996	[99]	Spain	OA, DTX-1, DTX-2, DTX-3, STXs, GTXs, neo-STXs	mixtures mainly of OA, DTX-2, GTXs and traces of STX
Villar Gonzalez et al., 2006	[100]	Spain	OA, DTX-1, DTX-2, DTX-3, SPX-1	mixtures of OA, DTX-2 and traces of SPX-1
Villar Gonzalez et al., 2007	[101]	Spain	OA, DTX-1, DTX-2, PTX-1, PTX-2, PTX-2 sa, AZA-1, YTX, SPX-1	OA/PTX-2sa and OA/PTX-2sa/SPX-1
de la Iglesia et al., 2009	[102]	Spain	PTX-6, YTX, 45-OH-YTX	mixtures of PTX-6 and YTXs
Rodriguez et al., 2015	[103]	Spain	OA, DTX-1, DTX-2, PTX-1, PTX-2, AZA-1, AZA-2, AZA-3, YTX, 45-OH-YTX, homoYTX, 45-OH-homoYTX	YTX/OA and OAs/YTX and YTXs/OA/PTX-2
García-Altares et al., 2016	[104]	Spain	OA, DTX-1, DTX-2, PTX-1, PTX-2, AZA-1, AZA-2, AZA-3, GYM, SPX-1, YTX, 45-OH-YTX, homoYTX, 45-OH-homoYTX	mixtures of OA and PTX-2
Stobo et al., 2005	[105]	UK	OA, DTX-1, DTX-2, PTX-1, PTX-2, AZA-1, AZA-2, AZA-3, YTX, 45-OH-YTX, homoYTX, 45-OH-homoYTX	YTX/OA and OA/AZA-1 and OA/YTX/PTX-2 and OA/PTX-2 and OA/YTX
Stobo et al., 2008	[106]	UK	OA, DTX-1, DTX-2, DTX-3, PTX-1, PTX-2, AZA-1, AZA-2, AZA-3, YTX, 45-OH-YTX, homoYTX, 45-OH-homoYTX	mixtures of OA, DTXs, PTXs and DA
Madigan et al., 2006	[107]	Australia	OA, PTX-2, GYM, YTX, DA	PTX-2s/OA
Takahashi et al., 2007	[108]	Australia	OA, DTXs, PTX-2, PTX-2 sa, GYM, DA	GYM/DA/PTX-2 and PTX-2s/OA/DA/GYM and PTX-2/OA
Ajani et al., 2017	[109]	Australia	OA, PTX-2, GYM, YTX, DA	PTX-2s/OA
MacKenzie et al., 2002	[110]	New Zealand	OA, DTX-1, PTXs, AZA-1, GYM, YTX, 45-OH-YTX, homoYTX, DA	YTXs/OA/PTX-2s/GYM/DA
McNabb et al., 2005	[111]	New Zealand	OA, DTX-1, DTX-2, PTXs, AZA-1, AZA-2, AZA-3, YTXs, GYM, SPXs, DA	PTX-2s/OA/YTXs/GYM and DA/OAs/PTX-2 and OAs/GYM/PTX-2/AZA-1/YTX

AZAs: azaspiracids; DTXs: dinophysistoxins; GTXs: gonyautoxins; GYMs: gymnodimines; PnTXs: pinnatoxins; PTXs: pectenotoxins; SPXs: spirolides; STXs: saxitoxins; YTXs: yessotoxins.

**Table 4:** Contamination with phycotoxin mixtures in other matrices.

Authors	Area	Toxin Mixtures	Matrix	Ref.
Zamorano et al., 2013	Chile	OAs/PTX-2/AZA-1/YTX/STXs	Gastropods	[41]
García et al., 2015	Chile	STXs/OA/DTX-1	Gastropods	[43]
García et al., 2016	Chile	OAs/PTX-2/YTX and OAs/YTX	Gastropods	[44]
Ganal et al., 1993	Hawaii	OA/CTX	Fish	[112]
Fire et al., 2011	U.S.	OA/DA/PbTx-3	Bottlenose dolphin	[113]
Wang et al., 2015	U.S.	OA/DTXs/PTX-2	Bottlenose dolphin	[114]
Kim et al., 2012	Korea	OA/YTX	Gastropods	[115]
Lee et al., 2012	Korea	OA/YTX	Gastropods	[116]
MacKenzie et al., 2011	New Zealand	OA/PnTxs	Gastropods	[117]

**Table 5:** Summary of in vivo studies.

Ref.	Animal	Treatment	Toxin (mg/kg b.w.)	Results Toxins alone		Results Mixtures	
				Distribution in Internal Organs ^a,b^	Macro- and Micro-Scopical Examination	Distribution in Internal Organs	Macro- and Micro-Scopical Examination
Aasen et al., 2011 [118]	Female NMRI mice	single intake by gavage	YTX: 1 or 5 AZA-1: 200 YTX/AZA-1: 1/200 or 1/500	- Highest levels of AZA-1 found in stomach, duodenum and jejunum - Highest levels of YTX found in duodenum, jejunum, ileum and colon	YTX: no effects AZA-1: retention of material in the stomach and dilatation of the upper 1/3 of the small intestine with increased fluidity; contraction and bluntness of villi from duodenum, extension of cryptal compartments and extensive infiltration of neutrophils in lamina propria	- Enhanced levels of YTX and AZA-1 in stomach - Enhanced levels of YTX in duodenum, jejunum and colon - Reduced level of YTX in liver	No mixture effect
Aune et al., 2012 [119]	Female NMRI mice	single intake by gavage	OA: 0.6; 0.82; 0.9; 0.98 or 1.14 AZA-1: 0.42; 0.54; 0.6; 0.66 or 0.78 OA/AZA-1 *: LD_10_/LD_10_ or LD_50_/LD_10_	- Highest levels of OA in GI tract - Highest levels of AZA-1 in stomach	OA: dilatation of stomach; shortened villi in the duodenum and jejunum and infiltration of neutrophils in lamina propria AZA-1: severe increase amount of content in stomach and dilatation of small intestine; shortened villi in the duodenum and infiltration of neutrophils in lamina propria	lower level for both toxins	No mixture effect
Sosa et al., 2013 [120]	Female CD-1 mice	repeated intake for 7 days by gavage	YTX: 1 OA: 0.185 YTX/OA: 1/0.185	Not investigated	YTX: ultrastructural changes in cardiomyocytes/OA: inflammation of the forestomach submucosa and ultrastructural changes in cardiomyocytes	Not investigated	No mixture effect

^a^ Brain, heart, lungs, thymus, liver, spleen, kidneys, stomach, small intestine (duodenum, middle and lower jejunum) and colon. * Lethal doses (LD) were estimated from individual toxin experiments. ^b^ Brain, heart, lungs, thymus, liver, spleen, kidneys, stomach, small intestine (duodenum, middle and lower jejunum) and colon.

**Table 6:** Summary of the study by Ferron et al., 2016 [123].

Cell Model	Treatment	Endpoint	Toxin Mixture (nM)		Mixture Effect
			Mixture	Molar Ratio *	
Caco-2	24-h incubation	Neutral red uptake	AZA-1/YTX	1:0.8	additive
				1:1.3	synergistic
				1:2.4	
				1:3.6	
			AZA-1/OA	1:51	antagonistic
				1:27.2	
				1:15.3	
				1:8.2	
			YTX/OA	1:26.5	antagonistic
				1:14.1	additive
				1:7.9	
				1:4.2	
Human intestinal epithelial crypt-like HIEC	24-h incubation	Neutral red uptake	AZA-1/YTX	1:0.8	synergistic
				1:1.3	
				1:2.4	
				1:3.6	additive
			AZA-1/OA	1:51	antagonistic
				1:27.2	additive
				1:15.3	
				1:8.2	antagonistic
			YTX/OA	1:26.5	synergistic
				1:14.1	antagonistic
				1:7.9	additive
				1:4.2	

* Molar ratios were based on IC_50_ values established for each toxin alone (OA: 78.52 nM, AZA-1: 4.03 nM and YTX: 4.08 nM).

## Supplementary Materials

The following are available online at http://www.mdpi.com/1660-3397/16/2/46/s1: Table S1: Calculation of ratio mixtures for each publication from the case study.
